# Large exchange bias enhancement and control of ferromagnetic energy landscape by solid-state hydrogen gating

**DOI:** 10.1038/s41467-023-43955-z

**Published:** 2023-12-21

**Authors:** M. Usama Hasan, Alexander E. Kossak, Geoffrey S. D. Beach

**Affiliations:** 1 Department of Materials Science and Engineering, Massachusetts Institute of Technology, Cambridge, MA 02139 USA; 2 Department of Materials and Metallurgical Engineering, Bangladesh University of Engineering and Technology, Dhaka, 1000 Bangladesh

**Keywords:** Magnetic properties and materials, Spintronics

## Abstract

Voltage control of exchange bias is desirable for spintronic device applications, however dynamic modulation of the unidirectional coupling energy in ferromagnet/antiferromagnet bilayers has not yet been achieved. Here we show that by solid-state hydrogen gating, perpendicular exchange bias can be enhanced by > 100% in a reversible and analog manner, in a simple Co/Co_0.8_Ni_0.2_O heterostructure at room temperature. We show that this phenomenon is an isothermal analog to conventional field-cooling and that sizable changes in average coupling energy can result from small changes in AFM grain rotatability. Using this method, we show that a bi-directionally stable ferromagnet can be made unidirectionally stable, with gate voltage alone. This work provides a means to dynamically reprogram exchange bias, with broad applicability in spintronics and neuromorphic computing, while simultaneously illuminating fundamental aspects of exchange bias in polycrystalline films.

## Abstract (web summary)

“Exchange bias” occurs in heterostructures of antiferromagnets and ferromagnetic materials, which biases the magnetization of the ferromagnet so that it exhibits a preferred direction. This phenomenon has proven critical for the development of a variety of spintronic devices. Here, Hasan et al demonstrate reversible control of exchange bias via solid-state hydrogen gating.

## Introduction

Exchange coupling in ferromagnet (FM)/antiferromagnet (AFM) bilayers can pin the FM in a preferred direction, a phenomenon known as exchange bias (EB)^1–7^. EB is widely used in spintronic devices such as spin-valves and tunnel junctions^2,8^ to passively bias the fixed layer. An emerging direction in spintronics research is to utilize AFMs as active components, which requires the ability to dynamically manipulate the AFM electrically^9–13^. Multiple paths to achieving this are being explored, including electric field gating^9,12,13^, current injection^11,14,15^, and ionic gating (i.e., magneto-ionics)^16,17^. The latter is unique because it can drive chemical changes in a diverse family of thin films via gate voltage^18–23^. It is a non-volatile^18,19^, low power^24^, and analog method^19,24^ capable of controlling a variety of magnetic properties, including magnetic anisotropy^18,23,25,26^ and exchange interactions^21,27–29^.

Most work on electrical control of EB uses magnetoelectrics^9,30,31^ or multiferroics^32,33^, where low temperatures^32,33^, field-cooling^9,30^, or complex growth processes^9,30–33^ are often required. Magneto-ionic approaches have also been reported^25,27,34–37^, but so far, have relied on redox of the FM^34^ or AFM^36^ in order to modulate the thicknesses or have targeted the FM to control properties such as anisotropy^25^ or magnetization^27^. These demonstrations, while promising, have only indirectly controlled the EB field ${H}_{EB}$, and not the underlying interfacial coupling strength.

Here we show that voltage-controlled solid-state protonation^18,21,27^ can trigger substantial, reversible changes in ${H}_{EB}$ by directly modulating the EB interaction energy. Under a small gate voltage ${V}_{G}$, EB can increase by >100% at room temperature (RT). We also demonstrate sub-millisecond and multi-state operation of the devices. We explain our results through a phenomenological EB model, which reveals that small modifications of the AFM grain rotatability can strongly modulate ${H}_{EB}$, and that hydrogen loading acts as an isothermal analog to field-cooling. Furthermore, we demonstrate electrical toggling of the FM energy landscape from a bi-stable to a mono-stable state at RT. This work provides a new look at interfacial EB and a means to control it dynamically, with potential impact on domain-wall-based devices, AFM spintronics, and brain-inspired computing.

## Results and discussion

### Measurement of EB and enhancement of EB by H-loading

**Fig. 1:**
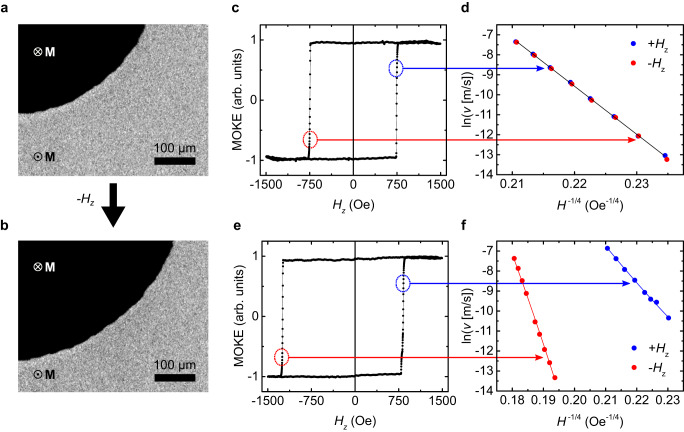
Exchange bias from DW creep. **a**, **b** Wide field MOKE images showing two domains separated by a DW where (**b**) is obtained from (**a**) after applying a ${-H}_{Z}$ pulse. **c** Hysteresis loop (${t}_{AFM}$ = 4.5 nm) showing no exchange bias. **d** ${{{{\mathrm{ln}}}}}\left(v\right)$ as a function of ${H}^{-1/4}$ for sample in (**c**) shows all data points lying on a single straight line. **e** Hysteresis loop (${t}_{AFM}$ = 12 nm) showing exchange bias. **f** ${{{{\mathrm{ln}}}}}\left(v\right)$ as a function of ${H}^{-1/4}$ for sample in (**e**); solid lines show fits with Eq. 1 with ${{H}_{EB}}^{}$ = −229 Oe. All measurements were performed at 20 ^o^C.

Most experiments focus on thin Co films with perpendicular magnetic anisotropy (PMA) in the heterostructure Ta/Pt/Co/Pt/Co_0.8_Ni_0.2_O/GdO_*x*_/Au (see Methods). The EB direction was set post-deposition by field cooling. Domain wall (DW) creep dynamics were exploited to extract ${H}_{EB}$. DW motion depends sensitively on local properties and, unlike hysteresis loops, is independent of the properties of nonlocal nucleation sites^38^. A magneto-optic Kerr effect (MOKE) microscope was used to image field-driven domain expansion (Fig. 1a, b) to extract the creep velocity, ${v}_{DW}$ (see Methods). In the absence of EB (Fig. 1c), ${v}_{DW}$ is independent of driving field polarity (Fig. 1d), whereas finite ${H}_{EB}$ (Fig. 1e) breaks this symmetry (Fig. 1f), as accounted for by the usual DW creep expression^39^:

1 $${v}_{DW}={v}_{0}\cdot{{{{{\rm{exp}}}}}}\left(-\frac{{E}_{b}}{{{{\rm{k}}}T}}{\left(\frac{{H}_{crit}}{{{{{{\rm{|}}}}}}H-{H}_{EB}{{{{{\rm{|}}}}}}}\right)}^{\frac{1}{4}}\right)$$

Here, ${H}_{crit}$ is the critical depinning field, ${E}_{b}$ is the disorder-induced barrier height, ${v}_{0}$ relates to attempt frequency, $k$ is the Boltzmann constant, $T$ is temperature, and $H$ is the applied field.

**Fig. 2:**
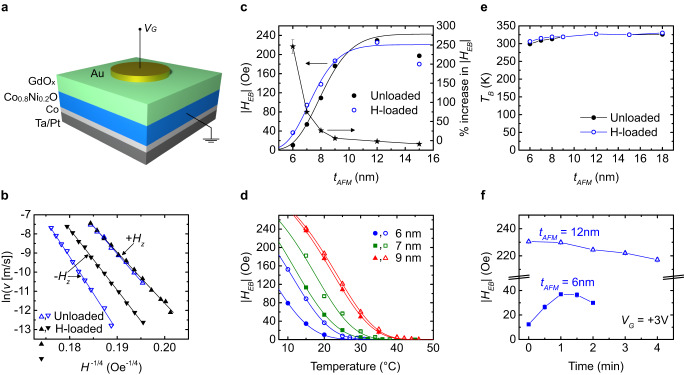
Effect of H-loading in Co_0.8_Ni_0.2_O. **a** Schematic of the device used for gating. **b** DW creep for ${t}_{AFM}$ = 7 nm in the unloaded and H-loaded (${V}_{G}\,$ = +2 V for 18 min) states at 20 °C. Solid lines are fits to Eq. 1, which gives ${H}_{EB}$ around −54 and −92 Oe for the unloaded and H-loaded states, respectively. **c** EB in the unloaded and H-loaded states as a function of AFM thickness at 20 °C; the right axis shows the percent change in EB. **d** Temperature dependence of EB for three AFM thicknesses where the solid (open) points represent the unloaded (H-loaded) state. Continuous solid lines in (**c**) and (**d**) are calculated from the model. **e** Blocking temperature in the unloaded and H-loaded states as a function of AFM thickness. **f** EB as a function of gating time at 20 °C. Error bars in ${H}_{EB}$ (**c**, **d**, **f**) are smaller than the symbols and represent the standard error of fitting of data, such as in (**b**) with Eq. (1). Error bars in (**c**) for the right axis are the errors propagated from the corresponding ${H}_{EB}$ values.

Figure 2a schematically illustrates our device structure. When positive ${V}_{G}$ exceeding a threshold is applied to the Au electrode, atmospheric water vapor dissociates and H^+^ is injected into the layers beneath as has been established elsewhere^18,22,27^. The influence of H gating was probed by measuring DW creep within the gated region, with exemplary data in Fig. 2b. The ${V}_{G}$-induced shift between the ${v}_{DW}(+H)$ and ${v}_{DW}(-H)$ branches signifies an increase of ${H}_{EB}$, quantified by fitting to Eq. (1). The effect is largely reversible under ${V}_{G} < 0$ (see Supplementary Information (SI), Fig. S2), which extracts the already injected H. Experiments show that ${H}_{EB}$ enhancement only occurs under ${V}_{G}\gtrsim$ 1 V (see SI, Section 11 and Fig. S13a) in humid atmospheres; no significant changes occur under positive bias in vacuum, and changes under negative bias are only seen if ${V}_{G}\gtrsim$ 1 V is first applied in a humid atmosphere (see SI, Sec. 1). These observations confirm that the primary effect of ${V}_{G}$ is due to H-injection/removal and that other electrochemical or electric-field effects are negligible. A small reduction (<20%) in PMA is observed with H-loading (SI, Section 10) in accordance with previous work, but net PMA is retained under the present conditions^18,23,25^.

Similar measurements were performed for devices with a range of AFM thickness, $${t}_{AFM}$$. Figure 2c, d shows the dependence of $${H}_{EB}$$ on $${t}_{AFM}$$ and on $$T$$, respectively, for the virgin state and after H loading. $${H}_{EB}$$ varies strongly with $${t}_{AFM}$$ when $${t}_{AFM}\, \lesssim \,$$10 nm (Fig. 2c) and vanishes for $${t}_{AFM} < $$ 6 nm at $$T$$ = 20 °C in the virgin state. The $${t}_{AFM}$$-dependent reduction in the isothermal $${H}_{EB}$$ coincides with a reduction in blocking temperature, $${T}_{B}$$, which is the temperature at which $${H}_{EB}$$ vanishes (Fig. 2d), generally lower than the Néel temperature (~335 K for bulk Co_0.8_Ni_.0.2_O, see SI, Section 3). H loading has a pronounced influence on EB in the low-$${t}_{AFM}$$ regime, where the threshold thickness for finite EB at RT decreases and $${H}_{EB}$$ can more than double (Fig. 2c). By contrast, at larger $${t}_{AFM}$$, $${V}_{G}$$ generates a modest decrease of $${H}_{EB}$$. The effect of $${V}_{G}$$ grows as $$T$$ is reduced below $${T}_{B}$$ (Fig. 2d), and for $${t}_{AFM}$$ = 6 nm, we observe a remarkable increase $$\Delta {H}_{EB} > $$ 70 Oe at the lowest $$T$$ measured here. The $${V}_{G}$$-induced change in $${T}_{B}$$ is small (Fig. 2e and Fig. S3b) and does not adequately account for the change in $${H}_{EB}$$. Moreover, the temporal evolution of $${H}_{EB}$$ during gating is complex, first increasing then decreasing in the low-$${t}_{AFM}$$ regime, but only monotonically decreasing for larger $${t}_{AFM}$$ (Fig. 2f).

We have observed similar EB enhancement in several Co_*x*_Ni_1−*x*_O compositions (see SI, Section 4), indicating the generality of the effect in these systems and enabling further tunability in ${T}_{B}$ by selecting the Co/Ni ratio in the AFM (see Fig. S3a). The effect of H in these systems cannot be explained by previously reported effects such as redox-induced changes in effective layer thicknesses or H-induced changes in the Co saturation magnetization ${M}_{S}$. Interfacial redox under ${V}_{G} \, > \, 0$ would chemically reduce the metal/oxide^18,36^, increasing the effective ${M}_{S}$ and/or decreasing ${t}_{AFM}$, both of which would lower ${H}_{EB}$. A *V*_G_-induced change in the Co/Ni ratio in the AFM, driven by selective redox can be ruled out since ${T}_{N}$ remains unchanged (see SI, Section 3) and the effect is also observed in pure NiO (see SI, Section 4). H-induced ${M}_{S}$ reduction^40^ would increase ${H}_{EB}$($\propto \frac{1}{{M}_{S}}$), but the fractional enhancement would be independent of ${t}_{AFM}$ and $T$, in contrast to experiments. Instead, the ${t}_{AFM}$-dependent nature of the susceptibility of ${H}_{EB}$ to ${V}_{G}$ suggests that the ${t}_{AFM}$-dependent AFM grain energetics play a central role.

### AFM grain rotatability and EB in polycrystalline films

Many models have been developed to treat EB theoretically^3–5,7^, and ${H}_{EB}$ generally varies in proportion to the interfacial exchange energy density, ${J}_{{Ex}}$. However, in polycrystalline AFMs, the collective behavior of the AFM grain ensemble is more important than the detailed coupling mechanisms at the interface^41–43^, and only a fraction of AFM grains contribute to EB. Those with anisotropy energy barrier ${K}_{AFM}V\, \lesssim \, {{{\rm{k}}}T}$ (anisotropy constant ${K}_{AFM}$, grain volume $V$) are in the superparamagnetic regime and are thermally unstable. Amongst the remaining thermally stable subset of grains, only those whose Néel order remains unchanged upon FM magnetization reversal can impose a unidirectional bias field on the latter. We term these grains fixed as opposed to rotatable grains that undergo coupled rotation/reversal with the FM. Whether a thermally-stable grain of a certain size contributes to ${H}_{EB}$, i.e., whether it is fixed or rotatable, hence depends on ${J}_{{Ex}}$, which couples the FM and AFM order, and ${K}_{AFM}$, which inhibits the AFM order from rotating with the FM. As shown in SI section 5, for coherently rotating uncoupled grains, the size of the AFM grain ultimately determines its rotatability, with the key result being a critical AFM grain volume ${V}_{C}\propto {(\frac{{J}_{{Ex}}}{{K}_{AFM}})}^{2}$ above which the Néel order of an AFM grain is fixed, such that it can contribute to ${H}_{EB}$.

We observe the most pronounced effects of ${V}_{G}$ on ${H}_{EB}$ when ${t}_{AFM} \, \lesssim \,$  10 nm. In this regime, ${H}_{EB}$ depends strongly on ${t}_{AFM}$ (and hence AFM grain volume), and ${T}_{B}$ is significantly lower than ${T}_{N}$, which indicates that in this thickness regime, ${H}_{EB}$ is limited by the AFM grain rotatability. The experimental results (H-induced decrease in critical ${t}_{AFM}$ (Fig. 2c), increase in ${T}_{B}$ (Fig. S3b), and increase of ${H}_{EB}$ for $T < {T}_{B}$) imply that H-loading decreases the AFM grain rotatability. As we show below, these behaviors can be well-described through the model in SI Sec. 5.

Assuming a thickness-dependent log-normal AFM grain volume distribution^41,44,45^ (SI, Section 5), we tested the applicability of this model on different AFM oxides and it correctly predicted the very different anisotropies of Co-rich and Ni-rich AFM oxides (Table S2 and Fig. S5). The solid lines in Fig. 2c, d show predictions based on this model for the unloaded and H-loaded cases of Co_0.8_Ni_0.2_O assuming a voltage-induced reduction in ${V}_{C}$. It qualitatively reproduces the key experimental behaviors related to H-loading and reveals that a change in the ratio $\frac{{J}_{{Ex}}}{{K}_{AFM}}$ of <10% is sufficient to trigger a several-fold increase in ${H}_{EB}$ at low ${t}_{AFM}$, owing to the inherent nonlinearity of thin-film grain size distributions (see SI, Section 6). It also makes the paradoxical prediction that a reduction in ${J}_{{Ex}}$ can actually increase ${H}_{EB}$ if ${V}_{C}$ shifts so as to allow additional grains to contribute to EB. This would qualitatively explain the time-dependent data in Fig. 2f: at larger ${t}_{AFM}$, most grains already contribute to EB due to larger ${K}_{AFM}V$, and so the monotonic ${H}_{EB}$ decrease under H loading reflects a proportional decrease in ${J}_{{Ex}}$. At low ${t}_{AFM}$, however, the same ${J}_{{Ex}}$ reduction would increase the fraction of thermally stable grains that are fixed, increasing the net EB, and eventually reduce ${H}_{EB}$ as further reduction of ${J}_{{Ex}}$ continues to decrease the exchange field imposed by all AFM grains. We note that, the sharper the grain size distribution, the more dramatic the H-induced EB modulation should be. Indeed, for a device scaled down to the size of a single AFM grain, it should be possible to toggle the EB off and on by reducing ${V}_{C}$ from above the volume of the AFM grain to below it.

H-induced reduction of exchange interactions, both in the bulk^27^ and at an interface^21^ has been reported before, and a similar phenomenon may be occurring in our system. Modulation of anisotropy in the AFM hematite (α-Fe_2_O_3_) with hydrogen absorption has also been reported but was explained in terms of hydrogen-induced reduction of the cation (Fe^3+^ to Fe^2+^), which cannot occur in Co_x_Ni_1−*x*_O. However, hydrogen induced oxygen vacancy creation cannot be ruled out and this mechanism may play a part in modifying the relevant parameters. A mechanistic understanding of H-induced changes in anisotropy and interfacial exchange is an important topic of future work.

### Validation of phenomenological model

**Fig. 3:**
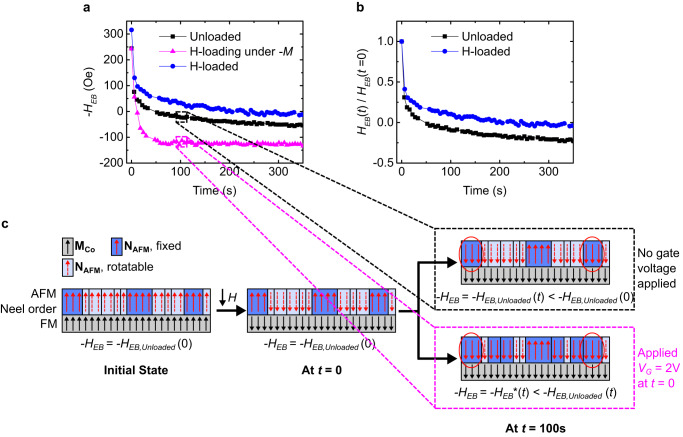
Experimental validation of the model. **a** EB as a function of cumulative time in the magnetization reversed state for the unloaded (black), during H-loading (magenta), and in the H-loaded states (blue) at 22 °C. **b** Normalized ${H}_{EB}$ for the unloaded and H-loaded devices, demonstrating that the EB reversal rate in the H-loaded state is lower than the unloaded state. **c** Schematic showing that the state of the AFM grains is different in the case of an unloaded device and a device where H is loaded during the measurement. A hundred seconds after beginning H-injection (right panel), there are more fixed AFM grains pointing down relative to the unloaded state, hence the lower EB. The red ellipses indicate grains that have undergone thermally activated reversal.

The data above suggest that in the low-${t}_{AFM}$ regime, the effect of H loading is analogous to field-cooling, namely, fixing of AFM grains against reversal in a configuration that biases the FM in the orientation it was in during this process, as well as making them more thermally-stable. The slight increase of ${T}_{B}$ after H-loading (Fig. S3b) directly supports this claim of increased stability. To further test this conjecture, we examined the influence of the FM orientation during gating on the ${V}_{G}$-induced change in ${H}_{EB}$. The experiments in Fig. 2 involved gating while the FM was parallel to the established EB direction (hereafter $+M$), resulting in an increase in ${{|H}}_{EB}|$ in the low-${t}_{AFM}$ regime, consistent with the field-cooling analogy. In Fig. 3, we explore the effect of gating on ${H}_{EB}$ when the FM orientation is anti-parallel to the established EB direction (hereafter $-M$). In the following, initialization refers to heating above ${T}_{B}$ and cooling under $+M$. For more details see Methods and SI, Section 8.

Figure 3a shows ${H}_{EB}$ versus cumulative time in the magnetization-reversed state ($-M$) for an unloaded device after initialization. ${{|H}}_{EB}|$ decreases with time, consistent with thermally activated reversal of AFM grains with $V > {V}_{C}$^43^, driven by the interfacial exchange field. Once reversed, these grains impose a bias field that prefers the $-M$ state, contributing to a net decrease and eventual sign reversal of ${H}_{EB}$.

This experiment was repeated after re-initializing ${H}_{EB}$, and then applying ${V}_{G} > 0$ to load and hold H (see Methods) while in the $-M$ state. Compared to the unloaded case, the ${H}_{EB}$ reversal rate is markedly enhanced, which implies that the number of AFM grains contributing to the opposite ${H}_{EB}$ grows at a faster pace. This could happen either through an H-induced increase in the thermal activation rate of AFM grain reversal, or an H-induced reduction of ${V}_{C}$ that fixes additional grains that contribute to EB with preference for the $-M$ state, analogous to field-cooling in the $-M$ state.

To discriminate between these possibilities, we took the H-loaded device, re-initialized its EB in the $+M$ state and tracked the evolution of ${H}_{EB}$ after subsequently reversing $M$. We find that ${{|H}}_{EB}|$ is higher when the H-loaded device is re-initialized in the $+M$ state compared to the unloaded case, revealing that the effect is independent of whether H is injected at RT after initialization (Fig. 2c) or if initialization is performed in the H-loaded state (Fig. 3a). The rate of ${H}_{EB}$ reversal in the $-M$ state is, in this case, substantially lower than in either of the cases above (Fig. 3a, b). If the presence of H increased the AFM grain reversal rate, the ${H}_{EB}$ reversal rate in this case should be the highest, in contrast with the experiment. These data indicate that H-loading decreases the rotatability of the AFM grains (Fig. 3b and Fig. S9) and that it is not simply the presence of H that determines the magnitude of ${H}_{EB}$, but the orientation of $M$ when it is introduced is also important. We conclude that H-loading is an isothermal analog to field-cooling: both processes decrease the rotatability of the AFM, resulting in the fixing of otherwise-rotatable AFM grains, which tend to imprint an EB field that prefers to maintain the orientation of $M$ present during the process.

### Toggling the FM between bi-stable and mono-stable states

**Fig. 4:**
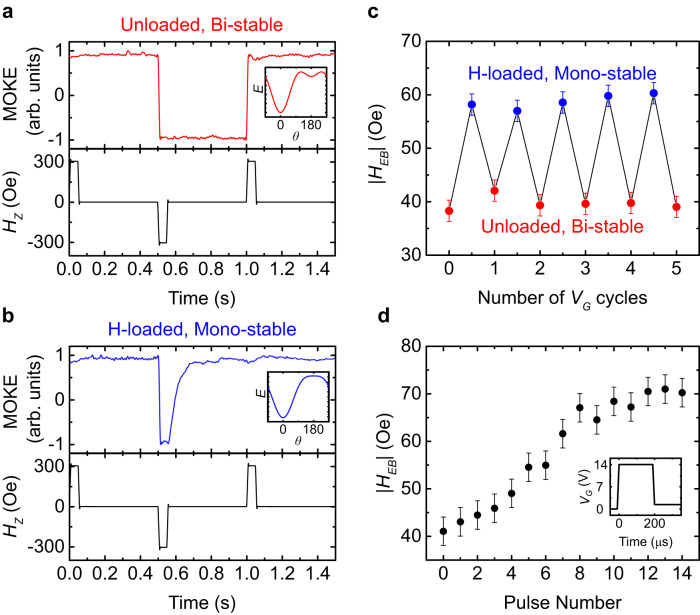
Toggling the FM between bi-stable and mono-stable states and high-speed operation. **a** MOKE signal of the unloaded state (top) as a field pulse sequence (bottom) is applied. **b** MOKE signal of the H-loaded state (top) as a field pulse sequence (bottom) is applied. **c** Reversible toggling between the bi-stable and mono-stable states by applying a positive bias (H-loaded) and negative bias (unloaded). **d** Multi-level EB enhancement enabled by 200 μs 14 V pulses (shown in inset). Measurements were performed at 22–23 °C. The insets in panels **a** and **b** show schematic magnetic energy landscapes based on a single-domain uniaxial ferromagnet with a unidirectional bias field in which two local minima exist (**a**) and only one energy minimum exists (**b**), corresponding to bistable and mono-stable states. Error bars represent an upper bound of the variation in ${H}_{EB}$ during the measurements.

So far, we focused on films with coercivity ${{H}_{C} \, > \, H}_{EB}$ to facilitate DW motion measurements under either field polarity. Here, we demonstrate ${V}_{G}$ toggling between ${{H}_{C} \, > \, H}_{EB}$ and ${{H}_{C} \, < \, H}_{EB}$ configurations, facilitating dynamic switching between bi-directional and unidirectional stability. We replaced the Co film with a low-${H}_{C}$ Co/Pt multilayer (see Methods and SI Section 9) and probed the stability of the $+M$ and $-M$ states before and after ${V}_{G}$ application. In Fig. 4a, the device was first initialized in the $+M$ state to induce a finite ${H}_{EB}$, and then the orientation of ${{{{{\bf{M}}}}}}$ was tracked versus time during a bipolar field pulse sequence. The temporal stability of both the $+M$ and $-M$ states is evident, implying that the field-cooled ${H}_{EB}$ is by itself insufficient to reverse ${{{{{\bf{M}}}}}}$. In Fig. 4b, the experiment was repeated after applying ${V}_{G} > 0$ in the $+M$ state. In this case, when the magnetization is reversed by an applied field pulse, it spontaneously reverts to $+M$ once the applied field is set to zero, indicating that ${H}_{EB}$ can overcome the switching energy barrier after H loading. In Fig. 4c, we show that ${H}_{EB}$ can be repeatedly toggled in this manner, allowing for reversible device toggling between bi-stable and mono-stable states. A small change (<10%) in ${H}_{C}$ occurs concurrently and appears to assist in the toggling (see SI, Fig. S11c).

Finally, by applying higher voltages, we demonstrate that a greater than 50% enhancement of the EB can be achieved in less than a millisecond (see SI, Section 11 and Fig. S13b) in these multilayer devices. Fast multi-level EB programming is also possible, as demonstrated in Fig. 4d with 14 V pulses of 200 μs width.

In summary, we have shown that EB can be markedly enhanced by solid-state protonation in Co/Co_*x*_Ni_1-*x*_O heterostructures in a reversible manner. We provided analytical and experimental evidence to suggest that the effect originates from new AFM grains becoming fixed isothermally due to hydrogen altering the AFM grain rotatability in a manner analogous to conventional field cooling. We further provide a proof-of-concept demonstration of bi-stable to mono-stable toggling of the FM energy landscape, which, together with sub-millisecond and multi-state operation, serves to establish the utility of this approach and suggests potential application in the development of low-power reprogrammable domain-wall based synapse/neurons, magnonic devices, and non-conventional computing platforms.

## Methods

### Sample preparation

All samples were grown via magnetron sputtering at room temperature. Samples corresponding to measurements in Figs. 1 and 2 had a layer structure Ta(4 nm)/Pt(3 nm)/Co(0.8 nm)/Pt(0.5 nm)/Co_0.8_Ni_0.2_O(*t*_*AFM*_ nm)/GdO_*x*_(22 nm)/Au(5 nm), where ${t}_{AFM}$ ranged from 4.5 to 30 nm. The nucleation density was low in these films, allowing for observation of a single DW propagating over extended distances. The samples for the EB drift experiment (Fig. 3) had the structure Ta(4 nm)/Pt(3 nm)/Co(0.35 nm)/Pt(0.5 nm)/Co_0.8_Ni_0.2_O(7 nm)/GdO_x_(22 nm)/Au(5 nm). Here, the lower Co thickness led to a much higher nucleation density (SI, Section 7) so that a conventional laser MOKE measurement yielded an accurate local measure of the EB field due to the presence of multiple nucleation events near the laser spot (<100 μm) and within the gated area. For demonstrating bi-stable to mono-stable configuration toggling (Fig. 4) we utilized a Co/Pt multilayer to reduce the coercivity (SI, Section 9), with a layer structure Ta(3 nm)/Pd(4 nm)/[Co(0.3 nm)/Pt(2 nm)]_2_/Co(0.35 nm)/Pt(0.5 nm)/Co_0.8_Ni_0.2_O(7 nm)/GdO_*x*_(22 nm)/Au(5 nm). In all cases, films were sputter deposited onto (100) thermally oxidized silicon substrates at room temperature. For metallic layers, an Ar pressure of 3 mTorr was used except for Au, which was grown under 3.5 mTorr Ar. In the case of the oxides, the AFM layers were grown by reactive sputtering under 1.6 mTorr Ar with ${P}_{{O}_{2}}$ of 0.07 mTorr. The GdO_x_ was deposited using radio-frequency sputtering with 3 mTorr Ar and ${P}_{{O}_{2}}$ of 0.7 mTorr.

The top Au gate electrodes (~300 μm diameter) were patterned using a shadow mask and served as a top electrode, whereas the continuous metal layers, including the conductive AFM, were used as the back gate. The Au electrode is discontinuous at this thickness (see ref. ^17^), which allows for a significant triple phase boundary region, i.e., regions where air, Au, and GdO_*x*_ are co-located and allows for H^+^ to be readily incorporated into the GdO_*x*_ under positive gate bias.

### Polar laser and wide-field MOKE measurements

MOKE measurements were performed using a 1 mW laser with a wavelength of 660 nm focused to a spot size of about 10 μm. Measurements were performed in the polar geometry, which is sensitive to out-of-plane magnetization. A commercial Peltier stage with a custom temperature control unit with an accuracy of 0.1 °C was used to control the substrate temperature in all experiments. The focused laser MOKE was integrated into a custom wide-field (WF-) MOKE microscope. In order to image domains, a sample was first saturated along the $+z$ direction and a reference image was acquired. Differential images were then obtained by subtracting the saturated image from subsequent images. Reverse domains were generated within the field of view by the application of a suitable nucleation field, and field pulses were used to increase or decrease their size using an electromagnet with a rise time of <30 ms. DW velocities were obtained by tracking the DW position as a function of field pulse duration.

As depicted in Fig. 3, ${H}_{EB}$ drifts with time when the FM is magnetized oppositely to the EB direction. To minimize this effect, the amount of time domains were left in the reversed state was minimized by using only short field pulses ( < 400 ms) to drive DWs, and after each displacement measurement, the sample was saturated in the original EB direction followed by a wait time that was sufficient to reverse the time-dependent drift in ${H}_{EB}$.

### Gate voltage application

A CuBe probe was used to make electrical contact with the top Au electrodes, and a second contact was made with the sufficiently conductive AFM that served as the back gate. A gate voltage between 2 and 3 V was used for both positive and negative biases for up to ~20 min depending on the specific AFM thickness and the device, unless otherwise noted. When the EB of a device was measured after a positive bias application, a holding voltage of +1.4 V to +1.5 V was used to prevent the spontaneous discharging of hydrogen^21,27^. Measurements were taken at 0 V when measuring the EB after a negative bias application.

### Modeling

The equation used to fit EB values was (SI, Section 5):

2 $${{{{{{\rm{|}}}}}}H}_{{{{EB}}}}{{{{{\rm{|}}}}}}=	\frac{{J}_{{{{int}}},0}}{{{{{{{\rm{e}}}}}}}^{\mu }}.\frac{{\left(1-\frac{T}{{T}_{N}}\right)}^{\frac{1}{3}}}{2{M}_{S}{t}_{{FM}}a{{{{{{\rm{e}}}}}}}^{\frac{3}{2}{\sigma }^{2}}}\\ 	\cdot \left(1-{{{{{\rm{erf}}}}}}\left(\frac{{{{{\mathrm{ln}}}}}\left(\frac{{J}_{{{{int}}},0}}{{{{{{{\rm{e}}}}}}}^{\mu }}.\frac{1}{2{K}_{{{{AFM}}},0}{a}{t}_{{{{AFM}}}}}{\left(1-\frac{T}{{T}_{N}}\right)}^{-\frac{2}{3}}\right)-{\sigma }^{2}}{\sqrt{2}\sigma }\right)\right)$$

where ${M}_{S}$ = 1400 emu/cm^3^ is the Co saturation magnetization, ${t}_{{FM}}\,$= 0.8 nm is the Co thickness, ${T}_{N}$ = 335 K is the Néel temperature of Co_0.8_Ni_0.2_O, ${K}_{{{{AFM}}},0}$ is the anisotropy constant of the AFM at $T$ = 0 K, ${t}_{{{{AFM}}}}$ is the AFM thickness and $a$ = 0.3 nm is the nearest neighbor cation distance in Co_0.8_Ni_0.2_O. ${T}_{N}$ was estimated from the measurements shown in Fig. 2e and was virtually unchanged upon gating (SI, Section 3). ${J}_{{{{int}}},0}$ is the exchange interaction (units of energy) at the interface between a single spin of the AFM and a single spin of the FM, at $T$ = 0 K. $\mu$ and $\sigma$ are the mean and standard deviation, respectively, of the log-normal distribution of the grain sizes ($D$).

${{J}_{{{{int}}},0}}$ is related to ${J}_{{Ex}}$ by

$${J}_{{Ex}}\propto \frac{{J}_{{{{int}}},0}}{{Da}}$$

In modeling the gate voltage-induced shift in ${H}_{EB}$, we fixed ${K}_{{{{AFM}}},0}$ and $\sigma$ and allowed $\frac{{J}_{{{{int}}},0}}{{{{{{{\rm{e}}}}}}}^{\mu }}$ to vary. See SI Sec. 5 and Table S3 for the full derivation and tabulated values of the fitted parameters.

### EB drift experiment

Before the start of the experiment, **M** was set in the same direction as the original field-cooling direction ($+M$ state). The acquisition time for one hysteresis loop was 0.5 s and there was a gap of at least 5 s between subsequent measurements during which the FM was kept in the $-M$ state. The time axis in Fig. 3a represents the time at which the hysteresis loops were acquired. Between the measurement of different states, the device was reset or re-initialized by field-cooling in $+{H}_{Z}$ from ~60 °C. For the measurement where gating was performed during the drift experiment, after H loading at ${V}_{{G}}$  = +2 V, a holding voltage of ${V}_{{G}}$  = +1.5 V was applied to prevent dehydrogenation^21,27^ during the remaining time of data acquisition, during the re-initialization process, and during subsequent measurement of the hydrogen loaded state.

### Supplementary information

Supplementary Information

Peer Review File

## Data Availability

The data that support the findings of this study are available from the corresponding author upon reasonable request.
