# Supplementary material for: Large exchange bias enhancement and control of ferromagnetic energy landscape by solid-state hydrogen gating
Source: Nat Commun. 2023 Dec 21;14:8510. doi: 10.1038/s41467-023-43955-z (PMC10740009; doi:10.1038/s41467-023-43955-z)
Supplement: Supplementary file 1 — Supplementary Information [file 41467_2023_43955_MOESM1_ESM.docx]

**Supplementary Information**

**Large exchange bias enhancement and control of ferromagnetic energy landscape by solid-state hydrogen gating**

M. Usama Hasan^1,2^, Alexander E. Kossak^1^ and Geoffrey S. D. Beach^1^*

*^1^Department of Materials Science and Engineering, Massachusetts Institute of Technology, Cambridge, Massachusetts 02139, USA*

*^2^Department of Materials and Metallurgical Engineering, Bangladesh University of Engineering and Technology, Dhaka 1000, Bangladesh*

**1. Effect of atmospheric condition and negative bias on virgin devices:**

A device with high nucleation density (see section 7) with the structure Ta(4 nm)/Pt(3 nm)/Co(0.35 nm)/Pt(0.5 nm)/Co_0.8_Ni_0.2_O(7 nm)/GdO_x_(22 nm)/Au(5 nm) was used to assess the effect of negative bias and atmospheric conditions. Data were collected from hysteresis loop measurement using MOKE.

Fig. S1a shows the ineffectiveness of a negative bias in causing any significant change in EB. On the other hand, Fig. S1c shows that EB enhancement upon positive bias application is highly dependent on the moisture content: in vacuum there is no effect while the higher the relative humidity, the higher is the EB enhancement rate. These together prove that hydrogen sourced from atmospheric water splitting, which can only occur at positive bias, is injected into the films and causes the observed effects. We do not fully understand the behavior of the coercivity in these samples.


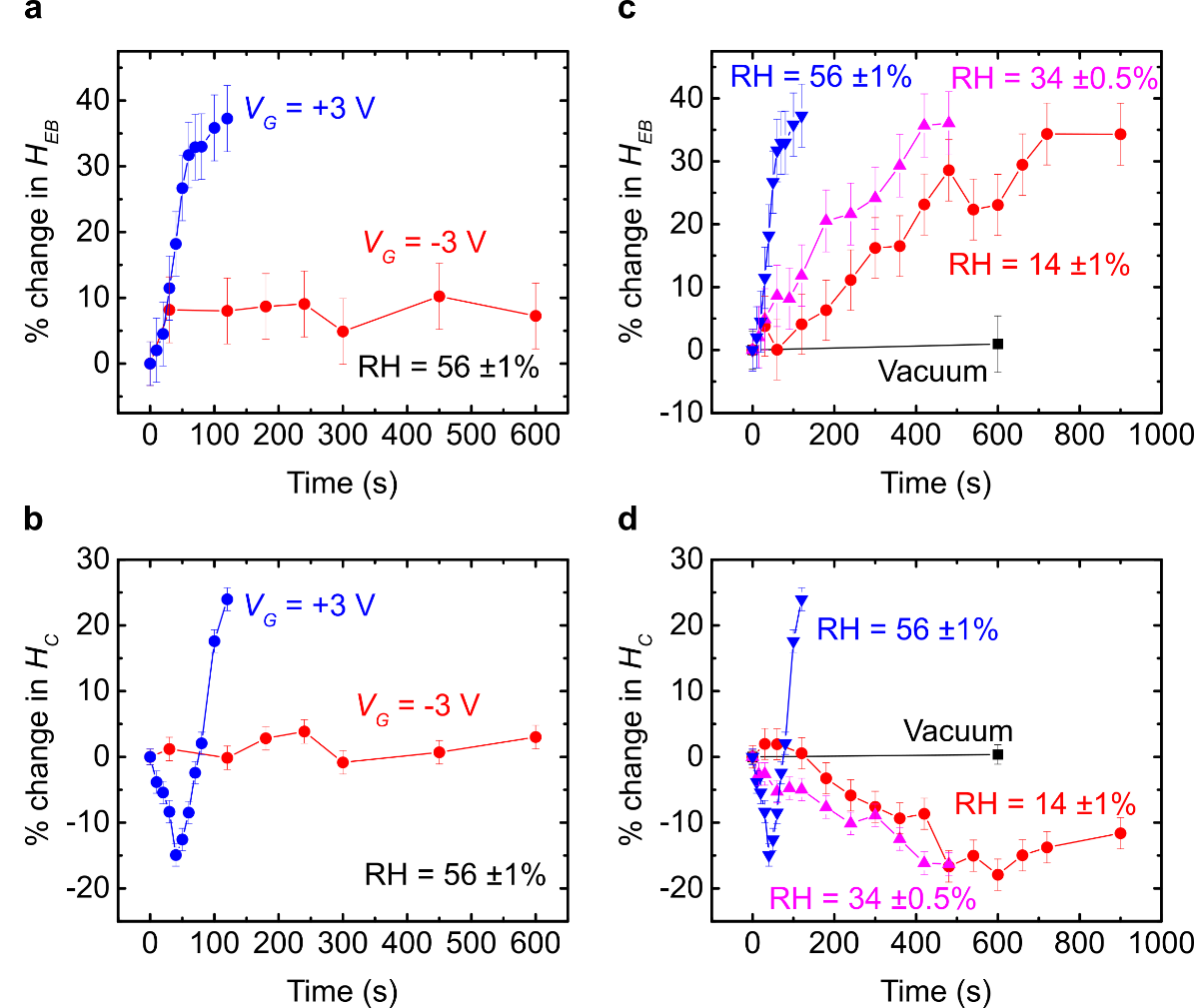


**Figure S1.** Effect of positive and negative bias in virgin devices on EB (**a**) and $H_{C}$ (**b**) as a function of time. Effect of relative humidity (RH) for $V_{G}$ = +3 V on EB (**c**) and $H_{C}$ (**d**) as a function of time. Error bars are derived from typical variation observed during hysteresis loop measurement.

**2. Effect of negative bias on H-loaded device:**


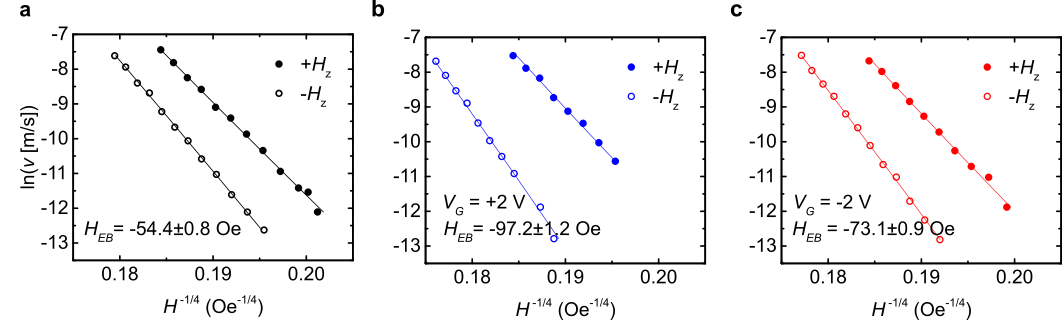


**Figure S2.** Domain wall creep for unloaded (**a**), H-loaded (**b**) and H-extracted (**c**) states. (**a**) and (**b**) is the same data as shown in Fig. 2b in the main text. Solid lines are fits with Eq. (1).

**3. Estimation of Neel temperature and nearest neighbor distance of AFM:**

Neel temperature of polycrystalline AFM was estimated from blocking temperature data as a function of AFM thickness. Fig. S3a depicts such data for three different oxide compositions: Co_x_Ni_1-x_O where x = 0.9, 0.8 and 0. The data was fitted with the equation^1^,

| $\frac{T_{N}-T_{B}}{T_{N}}= \left( \frac{J_{int, 0}}{{2K}_{AFM,0}{aD}_{max}t_{AFM}} \right)^{\delta}$ | (S1) |
| --- | --- |

where $T_{N}$ is the Neel temperature, $T_{B}$ is the blocking temperature, $J_{int, 0}$ is the interfacial interaction energy between a spin of the AFM and a spin of the FM at 0 K, $K_{AFM,0}$ is the anisotropy constant of the AFM at 0 K, $t_{AFM}$ is the thickness of the AFM, $a$is the nearest-neighbor distance in the AFM and $D_{max}$ is the size of the largest grain. For our case where the Curie temperature of the FM is much higher than the Neel temperature of the AFM, $\delta$ can be taken to be 3/2^1^. Fig. S3a shows the fits (solid lines) obtained with $T_{N}$ and $C=\frac{J_{int, 0}}{{2K}_{AFM,0}{aD}_{max}}$ as the fitting parameters. Table S1 tabulates the relevant parameters for the different oxides. The $T_{N}$ values are used later for the modelling. Fig. S3b shows the data of Fig. 2e from the main text for the unloaded and H-loaded case for Co_0.8_Ni_0.2_O. It is apparent that gating has a negligible effect on $T_{N}$ as both curves approach the same saturation point, but there is a slight but systematic increase in $T_{B}$ with H-loading, where the difference between the H-loaded and unloaded states increases as AFM thickness decreases.


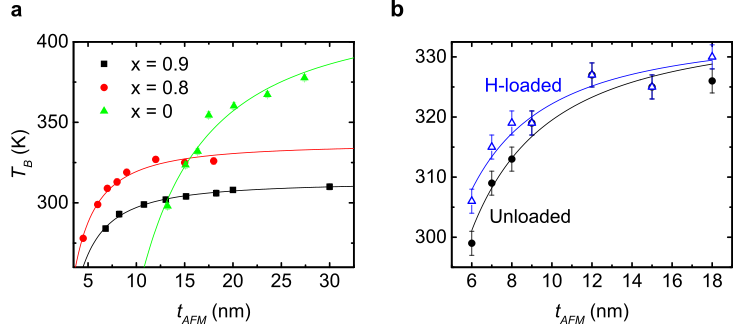


**Figure S3. a,** $T_{B}$ as a function of AFM thickness for Co_x_Ni_1-x_O with x given in the legend. **b**, $T_{B}$ as a function of AFM thickness for Co_0.8_Ni_0.2_O in the unloaded and H-loaded state. Solid lines are fits with Eq. S1. Error bars represent standard error of fitting of domain wall creep data to Eq. (1).

**Table S1.** Parameters obtained from fitting data in Fig. S3a,b to Eq. S1

| **Composition** | $\boldsymbol{T}_{\boldsymbol{N}}$  **(K)** | $\boldsymbol{C}$  **(cm)** |
| --- | --- | --- |
| Co_0.9_Ni_0.1_O | 313 ± 0.5 | (1.37 ± 0.03) x10^-7^ |
| Co_0.8_Ni_0.2_O | 335.4 ± 2.2 | (1.31 ± 0.09) x10^-7^ |
| NiO | 421 ± 7.3 | (5.68 ± 0.3) x10^-7^ |
| Co_0.8_Ni_0.2_O (H-loaded) | 334.6 ± 1.8 | (1.11 ± 0.08) x10^-7^ |

For calculating nearest neighbor distance ($a$), we need the lattice parameters. The lattice parameters for the end members of our composition series are 0.426 nm for CoO^2^ and 0.417 nm for NiO^3^ which are very close to one another. The nearest neighbor distance in FCC crystals is simply (√2/2 x lattice parameter) which we take to be 0.3 nm for all compositions.

**4. H-loading in other AFM compositions:**

Fig. S4 shows that H-loading in AFM with compositions Co_0.9_Ni_0.1_O and NiO also causes EB enhancement in a way very similar to that of the Co_0.8_Ni_0.2_O composition that is the focus of the main text. This demonstrates that H-loading is a viable way for EB modulation in the entire Co_x_Ni_1-x_O family of AFMs and is not restricted to a specific AFM or a specific sample. All EB values for these samples were extracted using domain wall creep measurements.

It should be noted that, for samples with NiO, the H-loading is spontaneous (i.e. no gate voltage is required) which is consistent with published work reporting that in a NiO/H_2_ battery cell, hydrogen is absorbed when discharging, i.e. is spontaneous ^6^.


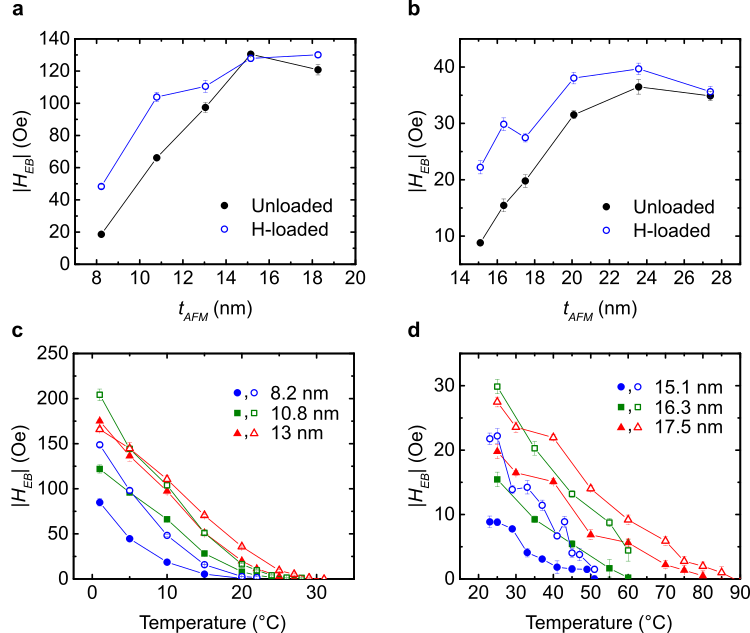


**Figure S4. H-loading in different AFM compositions.** EB as a function of AFM thickness for Co_x_Ni_1-x_O with x = 0.9 (**a**) and 0 (**b**) showing both the unloaded and H-loaded case. The temperature was 10 ^o^C and 25 ^o^C in (**a**) and (**b**) respectively. EB as a function of temperature for Co_x_Ni_1-x_O with x = 0.9 (**c**) and 0 (**d**). Solid (open) symbols represent unloaded and H-loaded states respectively. The legends in (**c**,**d**) indicate $t_{AFM}$. Error bars represent standard error of fitting of domain wall creep data to Eq. (1).

**5. Modelling exchange bias as a function of temperature and AFM thickness:**

For an isolated uniaxial AFM grain of volume $V$, where the Neel vector (**N**) is pointing along one of the easy directions, the energy barrier to a coherent reversal of its spins is $K_{AFM}\times V$, where $K_{AFM}\times V$ is the anisotropy energy of the AFM in units of energy/volume. Suppose now that there is a uniaxial FM in contact with the AFM that has its easy axis aligned with the AFM’s. There will be an interfacial exchange interaction between the AFM and the FM. If **N** and the FM’s magnetization **M** is aligned parallel, assume the energy of the system is lowest, ${-J}_{Ex}\times A$. If they are anti-parallel aligned the energy will be ${+J}_{Ex}\times A$. Here $A$ is the contact area between the AFM and FM and $J_{Ex}$ is the interfacial coupling strength in units of energy/area. This inequality of the energy between the two directions gives rise to exchange bias.

Assume the system is in the lowest energy state, i.e., the parallel condition. If a strong enough external field now reverses the **M** of the FM, the FM and AFM becomes unfavorably aligned (local minimum). The system will seek to go into the lowest energy state (global minimum) by reversing the AFM grain as well. The energy barrier for this to happen can be shown to be^4^,

$$\Delta E=\frac{{({2K}_{AFM}V-J_{Ex}A)}^{2}}{4K_{AFM}V}$$

However, if the interfacial coupling is strong enough, then the FM can force the AFM grain to rotate with it. If $J_{Ex}$ is continually increased from zero, the onset of this phenomenon will occur when there is no energy barrier between the local minimum and the global minimum, i.e., when

$$\Delta E=0$$

$$\to{2K}_{AFM}V-J_{Ex}A=0$$

| $\to\frac{V}{A}=\frac{J_{Ex}}{{2K}_{AFM}}$ | (S2) |
| --- | --- |

Assume that the AFM grains are columnar with grain size $D$ and thickness $t_{AFM}$. Furthermore, assume that the interfacial exchange interaction is due to random defects at the AFM interface^1^, which leads to an expression of $J_{Ex}$ that depends on the grain size as^1^,

| $J_{Ex}=\frac{J_{int}}{Da}$ | (S3) |
| --- | --- |

Here, $J_{int}$ is the exchange interaction strength between a single spin of AFM and a single spin of FM on either side of the interface in units of energy and $a$ is the nearest neighbor distance in the AFM. Plugging all of this into Eq. S2, we get,

$$\frac{\pi D^{2}t_{AFM}}{\pi D^{2}}=\frac{J_{int}}{{2K}_{AFM}Da}$$

$$\to D=\frac{J_{int}}{{2K}_{AFM}at_{AFM}}$$

Now plugging in the often-used power law temperature dependencies^1^ of $J_{int}$ and $K_{AFM}$, we get,

| $D_{C}=\frac{J_{int, 0}\left( 1-\frac{T}{T_{N}} \right)^{\frac{1}{3}}}{{2K}_{AFM,0}\left( 1-\frac{T}{T_{N}} \right)at_{AFM}}=\frac{J_{int, 0}}{{2K}_{AFM,0}at_{AFM}}\left( 1-\frac{T}{T_{N}} \right)^{- \frac{2}{3}}$ | (S4) |
| --- | --- |

where $T_{N}$ is the Neel temperature of the AFM. This size is the critical grain size $D_{C}$ as a function of temperature and AFM thickness– if a grain is bigger than this then the coupling is weak enough for the energy barrier to be non-zero and it can contribute to EB; if a grain is smaller than this the coupling strength becomes strong enough that the FM makes the AFM grain reverse along with it and no EB can manifest. This can also be viewed as a critical volume,

$$V_{C}=\frac{\pi D_{C}^{2}t_{AFM}}{4}=\frac{J_{int, 0}^{2}}{{16K}_{AFM,0}^{2}a^{2}t_{AFM}}\left( 1-\frac{T}{T_{N}} \right)^{- \frac{4}{3}}$$

which was the form alluded to in the main text.

In order to calculate the EB for a given temperature and AFM thickness, we use the basic formula for the EB field,

| $\vert H_{EB}\vert=\frac{J_{Ex, avg}}{M_{S}t_{FM}}$ | (S5) |
| --- | --- |

where $J_{Ex, avg}$is the exchange coupling (energy/area) averaged over all AFM grains, $M_{S}$ is the saturation magnetization of the FM and $t_{FM}$ is its thickness. $J_{Ex, avg}$ can be calculated as follows:

Eq. S3 gives the exchange coupling in energy/area for a grain of size $D$. Multiplying it with the area of the grain gives the coupling strength in units of energy. Now assuming that there is no intergranular exchange between AFM grains, we can simply sum up the contribution of all the grains to obtain the total coupling strength in units of energy,

$$\sum_{i=1}^{N} \frac{J_{int}}{D_{i}a}\cdot\frac{\pi D_{i}^{2}}{4}$$

If the individual AFM grain areas are much smaller than the area for which EB is being calculated, we can turn the sum into an integral and utilize a continuous probability density function to represent the grain size distribution. Assuming a log-normal distribution and making use of the fact that grains below $D_{C}$ cannot contribute to EB, we have,

$$\int_{D_{C}}^{\infty} \frac{J_{int}}{Da}\cdot\frac{\pi D^{2}}{4}\cdot f\left( D \right)dD$$

$$\to\int_{D_{C}}^{\infty} \frac{J_{int}}{Da}\cdot\frac{\pi D^{2}}{4}\cdot\frac{1}{D\sigma\sqrt{2\pi}}exp\left( -\frac{\left( \ln\left( D \right)-\mu\right)^{2}}{2\sigma^{2}} \right)dD$$

$$\to\frac{{\pi J}_{int}}{4a}\int_{D_{C}}^{\infty} \frac{1}{\sigma\sqrt{2\pi}}exp\left( -\frac{\left( \ln\left( D \right)-\mu\right)^{2}}{2\sigma^{2}} \right)dD$$

$$\to\frac{{\pi J}_{int}}{4a}{}_{D_{C}}^{\infty}\left[ \frac{\exp\left( \frac{\sigma^{2}}{2}+\mu\right)\mathrm{erf} \left( \frac{\ln\left( D \right)-\sigma^{2}-\mu}{\sqrt{2}\sigma} \right)}{2}+C \right]$$

$$\to\frac{{\pi J}_{int}}{4a}\left( \frac{\exp\left( \frac{\sigma^{2}}{2}+\mu\right)}{2}-\frac{\exp\left( \frac{\sigma^{2}}{2}+\mu\right)\mathrm{erf} \left( \frac{\ln\left( D_{C} \right)-\sigma^{2}-\mu}{\sqrt{2}\sigma} \right)}{2} \right)$$

$$\to\frac{{\pi J}_{int}}{8a}\exp\left( \frac{\sigma^{2}}{2}+\mu\right)\left( 1-\mathrm{erf} \left( \frac{\ln\left( D_{C} \right)-\sigma^{2}-\mu}{\sqrt{2}\sigma} \right) \right)=J_{total}$$

Here $\sigma$ and $\mu$ are the standard deviation and mean of the log-normal distribution respectively and erf() denotes the error function. We have made use of the fact that ln(∞)→∞ and erf(∞)→1, in line 5. This is the total energy for all the grains. $J_{Ex, avg}$ can be found by dividing by the total area of these grains,

$$J_{Ex,avg}=\frac{J_{total}}{A_{total}}=\frac{J_{total}}{\int_{0}^{\infty} \frac{\pi D^{2}}{4}f\left( D \right)dD}$$

$$\to J_{Ex,avg}=\frac{J_{total}}{\frac{\pi}{4}exp(2\sigma^{2}+2\mu)}$$

$$\to J_{Ex,avg}=\frac{\frac{J_{int}}{2a}\exp\left( \frac{\sigma^{2}}{2}+\mu\right)\left( 1-\mathrm{erf} \left( \frac{\ln\left( D_{C} \right)-\sigma^{2}-\mu}{\sqrt{2}\sigma} \right) \right)}{exp(2\sigma^{2}+2\mu)}$$

$$\to J_{Ex,avg}=\frac{J_{int}}{2a}\frac{\left( 1-\mathrm{erf} \left( \frac{\ln\left( D_{C} \right)-\sigma^{2}-\mu}{\sqrt{2}\sigma} \right) \right)}{exp\left( \frac{3}{2}\sigma^{2}+\mu\right)}$$

plugging this value in Eq. S5, and incorporating the temperature dependence of $J_{int}$, we get,

$${|H}_{EB}|= \frac{J_{int, 0} \left( 1-\frac{T}{T_{N}} \right)^{\frac{1}{3}}}{2M_{S}t_{FM}a}\cdot\frac{\left( 1-\mathrm{erf} \left( \frac{\ln\left( D_{C} \right)-\sigma^{2}-\mu}{\sqrt{2}\sigma} \right) \right)}{exp\left( \frac{3}{2}\sigma^{2}+\mu\right)}$$

plugging in the expression of $D_{C}$ from Eq. S4 and rearranging,

| ${\vert H}_{EB}\vert=\frac{J_{int, 0}}{e^{\mu}}.\frac{\left( 1-\frac{T}{T_{N}} \right)^{\frac{1}{3}}}{2M_{S}t_{FM}ae^{\frac{3}{2}\sigma^{2}}}\cdot\left( 1-\mathrm{erf} \left( \frac{\ln\left( \frac{J_{int, 0}}{e^{\mu}}.\frac{1}{2K_{AFM,0}at_{AFM}}\left( 1-\frac{T}{T_{N}} \right)^{- \frac{2}{3}} \right)-\sigma^{2}}{\sqrt{2}\sigma} \right) \right)$ | (S6) |
| --- | --- |

In order to test the validity of the model, we use this equation to fit the EB as a function of $t_{AFM}$ and $T$ for the three different oxides as shown in Fig. S5. Good qualitative fits are obtained by freely varying the parameters $\frac{J_{int, 0}}{e^{\mu}}$ , $K_{AFM,0}$ and $\sigma$ and using $M_{S}$= 1400 emu/cm^3^, $t_{FM}$ = 0.8 nm and $a$ = 0.3 nm. $T_{N}$ values from Table S1 were used for the respective oxides. A range of values for the parameters for a given oxide are obtained by fitting different datasets; these are summarized in Table S2. We find $K_{AFM,0}$ is over an order of magnitude smaller for NiO compared to the Co rich compositions which matches well with previously reported values^5^. It should be noted that the model cannot account for the peak and eventual dip in the EB as a function of AFM thickness.


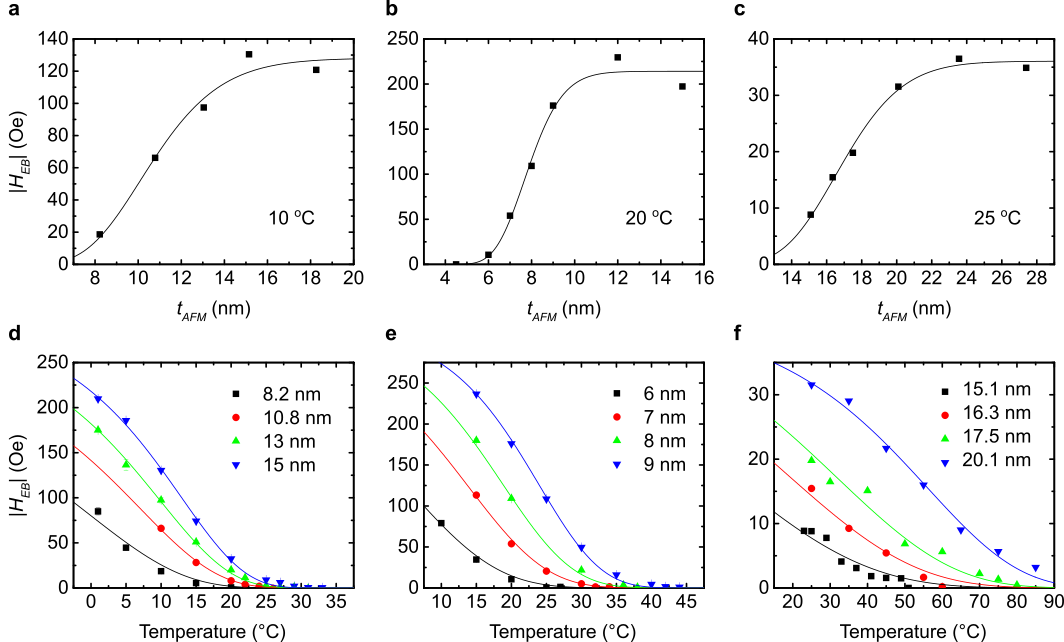


**Figure S5. Applicability of model to different AFM compositions.** EB as a function of AFM thickness for Co_x_Ni_1-x_O with x = 0.9 (**a**), 0.8 (**b**) and 0 (**c**) and EB as a function of temperature for Co_x_Ni_1-x_O with x = 0.9 (**d**), 0.8 (**e**) and 0 (**f**). The legends in (**d,e,f**) indicate $t_{AFM}$. Solid lines are fits with Eq. S6.

**Table S2.** Parameters obtained from fits to Eq. S5 of the data in Fig. S5

| **Composition** | $\boldsymbol{T}_{\boldsymbol{N}}$ **(from Table S1)**  **(K)** | $\frac{\boldsymbol{J}_{\boldsymbol{int, 0}}}{\mathbf{e}^{\boldsymbol{\mu}}}$  **(erg/cm)** | $\boldsymbol{K}_{\boldsymbol{AFM,0}}$  **(erg/cm^3^)** | $\boldsymbol{\sigma}$ |
| --- | --- | --- | --- | --- |
| Co_0.9_Ni_0.1_O | 313 ± 0.5 | (1.02 –1.85) x10^-9^ ±10% | (7.21 – 9.68) x10^4^  ±8% | 0.23 ±30% |
| Co_0.8_Ni_0.2_O | 335.4 ± 2.2 | (1.47 – 1.89) x10^-9^ ±8% | (1.19 – 1.41) x10^5^  ±5% | (0.14 – 0.16) ±17% |
| NiO | 421 ± 7.3 | (1.89 – 2.04) x10^-10^ ±10% | (4.13 – 4.31) x10^3^  ±7% | (0.16 – 0.17) ±10% |

**6. Modelling the effect of H-loading on EB:**

As mentioned in the main text, we propose that H-loading reduces the critical volume, $V_{C}$, below which AFM grains cannot contribute to EB. This can increase the number of grains contributing to the bias field in the low-$t_{AFM}$ regime, resulting in EB enhancement, while in the high-$t_{AFM}$ regime, most of the grains are already bigger than $V_{C}$, hence its reduction has no appreciable effect. This scenario is schematically represented in Fig. S6.


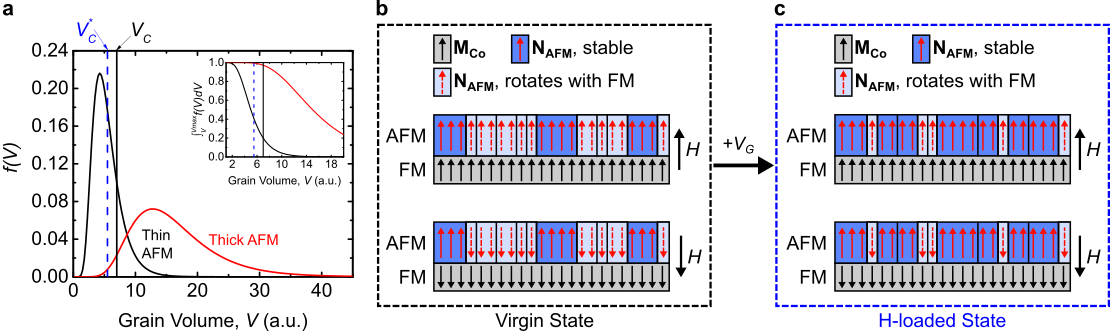


**Figure S6. Model to account for EB enhancement with H-loading**. **a,** Hypothetical distribution of grain volumes in a thin and thick AFM film. The black and blue vertical lines show the position of $V_{C}$ before and after gating, respectively. Inset plots integrated area under the curves in the limits $V$ to $V_{max}$. **b-c,** Schematic showing how the Neel vector (**N_AFM_**) can reverse (light colored arrows) with the FM magnetization (**M_Co_**) or be fixed (dark colored arrows) depending on the size of the AFM grain (grain boundaries shown as black lines). In the H-loaded state, some smaller AFM grains become fixed and increase the total fraction of grains that are contributing to EB.

**Table S3.** Parameters obtained from fits to Eq. S6 of the data in Fig. 2c,d of the main text. * indicates parameters for the H-loaded case

| **Dataset** | $\frac{\boldsymbol{J}_{\boldsymbol{int, 0}}}{\mathbf{e}^{\boldsymbol{\mu}}}$  **(erg/cm)** | $\left( \frac{\boldsymbol{J}_{\boldsymbol{int, 0}}}{\mathbf{e}^{\boldsymbol{\mu}}} \right)^{\boldsymbol{*}}$  **(erg/cm)** | $\boldsymbol{K}_{\boldsymbol{AFM,0}}$  **(erg/cm^3^)** | $\frac{\boldsymbol{V}_{\boldsymbol{C}}^{\boldsymbol{*}}}{\boldsymbol{V}_{\boldsymbol{C}}}$ | $\boldsymbol{\sigma}$ |
| --- | --- | --- | --- | --- | --- |
| **EB vs *t* at 20 ^o^C (Fig. 2a)** | (1.73 ± 0.06) x10^-9^ | (1.57 ± 0.05)  x10^-9^ | (1.36 ± 0.03) x10^5^ | 0.82 | 0.2 ± 0.014 |
| **EB vs *T* for** $\boldsymbol{t}_{\boldsymbol{AFM}}$ **= 6nm (Fig. 2c)** | (1.99 ± 0.14) x10^-9^ | (1.82 ± 0.13) x10^-9^ | (1.74 ± 0.10) x10^5^ | 0.84 | 0.12 ± 0.002 |
| **EB vs *T* for** $\boldsymbol{t}_{\boldsymbol{AFM}}$ **= 7nm (Fig. 2c)** | (2.21 ± 0.67) x10^-9^ | (2.03 ± 0.62) x10^-9^ | (1.8 ± 0.44) x10^5^ | 0.84 | 0.14 ± 0.018 |
| **EB vs *T* for** $\boldsymbol{t}_{\boldsymbol{AFM}}$ **= 9nm (Fig. 2c)** | (2.25 ± 0.06) x10^-9^ | (2.2 ± 0.06) x10^-9^ | (1.64 ± 0.04) x10^5^ | 0.96 | 0.17 ± 0.004 |

For quantitative predictions, a given set of virgin and H-loaded state EB values were simultaneously fitted with Eq. S6 where $K_{AFM,0}$ and $\sigma$ were forced to be the same in both states, while $\frac{J_{int, 0}}{e^{\mu}}$ was allowed to vary. The obtained values of the parameters for the cases shown in Fig. 2 in the main text are tabulated in Table S3.

**7. Magnetization reversal in film used for EB drift experiment:**

For experimental validation of the proposed model the EB drift experiment with/without gating was performed as shown in Fig. 3. For that experiment it was necessary to be able to probe the state of the device in short time intervals on the order of 5 seconds. A device structure where the Co layer was about half as thick as usual yielded much higher domain nucleation density in the films. Fig. S7 shows this domain structure evolving. The top Au electrodes were circular with around 300 μm diameter. With the nucleation density as shown in the figure, one electrode would easily cover multiple nucleation sites, making it possible to probe a device’s state with simple focused laser MOKE.


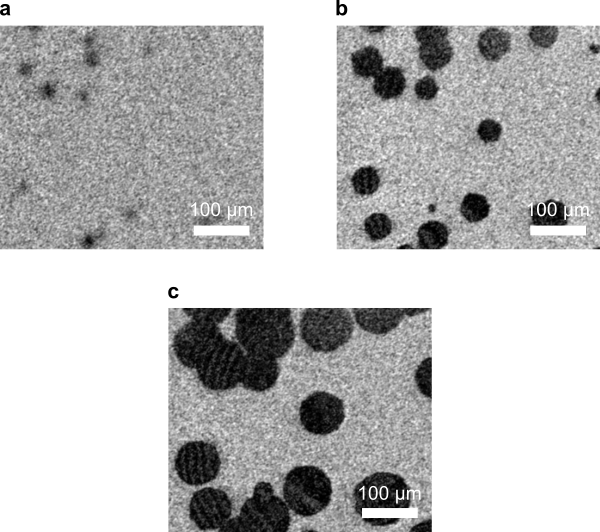


**Figure S7. Domain nucleation and growth in samples for EB drift experiment.** Differential MOKE images after application of 1 (**a**), 2 (**b**) and 3(**c**) external field pulse(s) of height $H_{Z}$ = -1117.6 Oe and width *t* = 200 ms. Light (dark) region corresponds to +**M** (-**M**).

**8. EB drift experiment:**

The top panel of Fig. S8 shows the same data as in Fig. 3a of the main text, but also includes the negative bias case. As expected, the negative bias case has no effect on the EB or its time evolution. For the H-loading or positive bias case, current induced temperature rise is not important since the power input is very low (< 0.6 μW), as depicted in the bottom panel of Fig. S8.

Fig. S9 shows evolution of normalized EB in the same sample as Fig. S8 and Fig. 3 as a function of cumulative time in the magnetization reversed state for the unloaded case at three different temperatures as well as the H-loaded case for 22 ^o^C. The thermally activated AFM grain reversal rate gets faster as temperature increases as evidenced by the quicker decay rate of EB at higher temperatures. In other words, the AFM grains become more thermally stable (less rotatable) as temperature decreases.


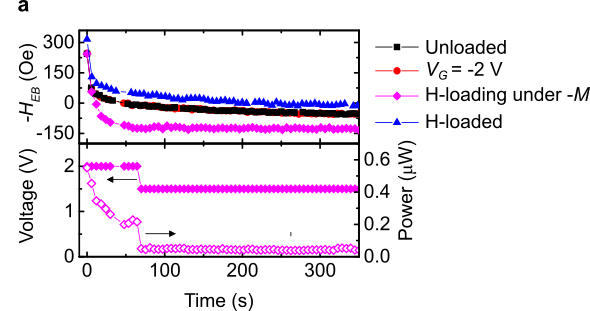


**Figure S8. EB drift experiment.** Top panel shows evolution of EB as a function of cumulative time in the magnetization reversed state, and bottom panel shows the voltage and power during the H-loading experiment.

On the other hand, the H-loaded state at 22 ^o^C is remarkably like the unloaded 18 ^o^C case, offering a direct proof that H-loading causes the rotatability of the AFM grains to go down.


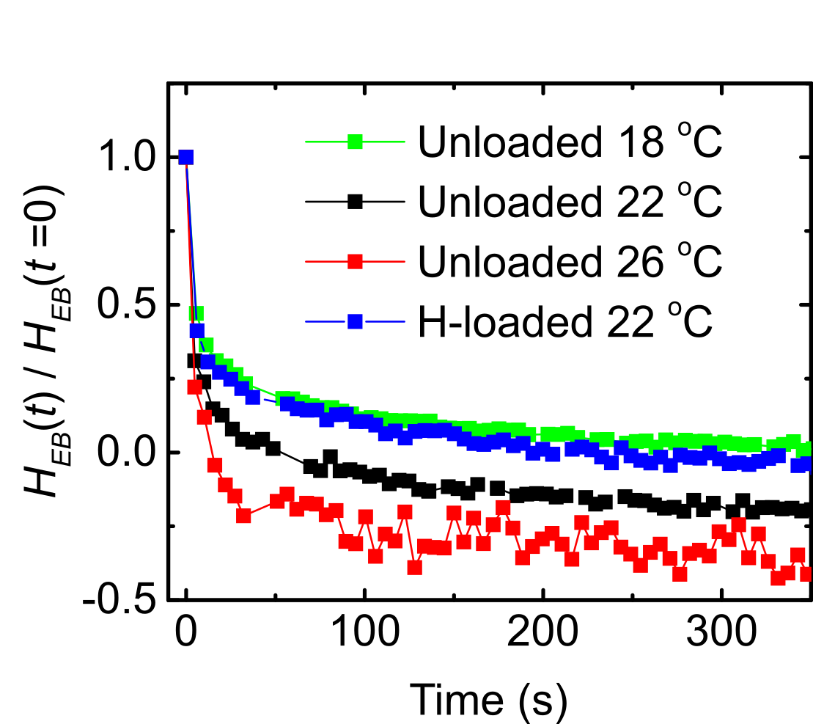


**Figure S9.** Evolution of EB (normalized to value at $t$ = 0) in the unloaded state as a function of cumulative time in the magnetization reversed state at different temperatures. The evolution of the H-loaded state is also shown for 22 ^o^C.

**9. Magnetization reversal and hysteresis loops in multilayer films:**

The multilayer films had very high nucleation density facilitating the use of MOKE in order to probe a device’s state rapidly. Exemplary images are shown in Fig. S10.

Full hysteresis loops were obtained for these films in addition to utilizing field pulses to interrogate the state of the device. Even though it is apparent from the field pulse experiments that the energy landscape of the devices can be toggled from a bi-stable state to a mono-stable state, this is not apparent from the hysteresis loops themselves.

Typically, a mono-stable state would be expected to be characterized by a hysteresis loop that is “full-shifted” i.e., where the coercive field is lower than the exchange bias field. However, the loops for our devices do not look like that (Fig. S11), the reason for which is the following.


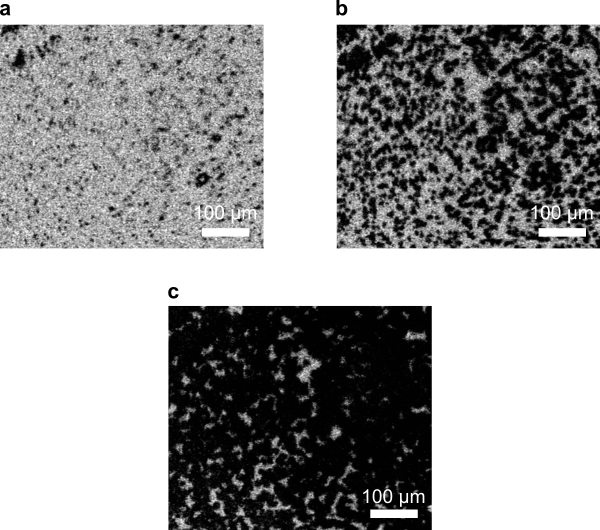


**Figure S10. Domain nucleation and growth multilayer samples.** Differential MOKE images after application of 1 (**a**), 2 (**b**) and 3(**c**) external field pulse(s) of height $H_{Z}$ = -76.2 Oe and width *t* = 150 ms. Light (dark) region corresponds to +**M** (-**M**)

First, there is a pronounced field sweep rate dependence of the switching fields. Domain nucleation is a thermally activated process, and thus, for a faster sweep rate, the switching happens at higher fields compared to a slower sweep rate. This effect causes the coercive field to go down as sweep rate is slowed down. Since this effect should not affect the exchange bias, one would want to minimize the measured coercive field by sweeping as slowly as possible, so that one can accurately assess whether the loop is “fully-shifted”. If this were the only effect in play, it would have been possible to show a hysteresis loop that starts out not “fully-shifted”, and becomes “fully-shifted” as it is gated. However, there is also the effect of EB drift in play.


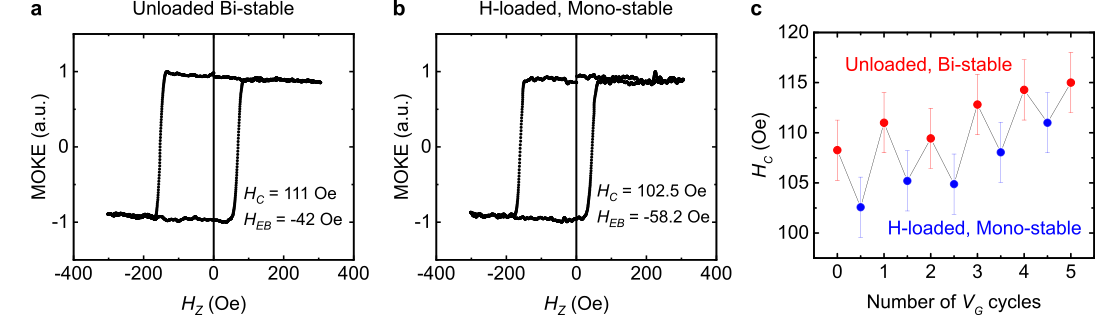


**Figure S11.** Typical hysteresis loops for multilayer films in the unloaded (**a**) and the H-loaded (**b**) states. **c,** shows the changes in coercivity as the device is toggled between bi-stable and mono-stable states. Error bars represent typical variation observed during hysteresis loop measurement.

EB drift causes the EB to reduce the longer a device is magnetized in the reversed direction. If the field sweep rate is lowered, the time that the device spends in the reversed state grows longer which decreases the exchange bias. Hence, if one lowers the sweep rate in hopes of lowering the coercivity, the EB also reduces due to this effect. Ultimately, for our devices there is no sweep rate that allows hysteresis loops to capture the toggling between bi-stable and mono-stable states.

Fig. S3c also shows that small changes (<10 %) in the coercivity happens concurrently with the EB, which assist in toggling between the bi-stable and mono-stable states.

**10. Anisotropy of multilayer films:**

The out-of-plane anisotropy of the films were estimated by measuring the out-of-plane MOKE contrast as a function of in-plane field. Fig. S12a shows the measured data for the virgin case as well as after negative bias and positive bias application. The negative bias case shows no change while the positive bias or H-loaded state reveals a slight reduction in the in-plane saturation field, $H_{K}$. The data were fitted with the equation,

$m= \sqrt{\left( 1-\left( \frac{H}{H_{K}} \right)^{2} \right)}$ (S7)

where m is the out-of-plane MOKE contrast and $H$ is the applied in-plane field. The fitted $H_{K}$ values were 12.5 ± 0.15 kOe, 12.4 ± 0.44 kOe and 10.2 ± 0.18 kOe for the virgin, negative bias and positive bias case respectively. Fig. S12b shows the corresponding coercivity and EB for the different states.


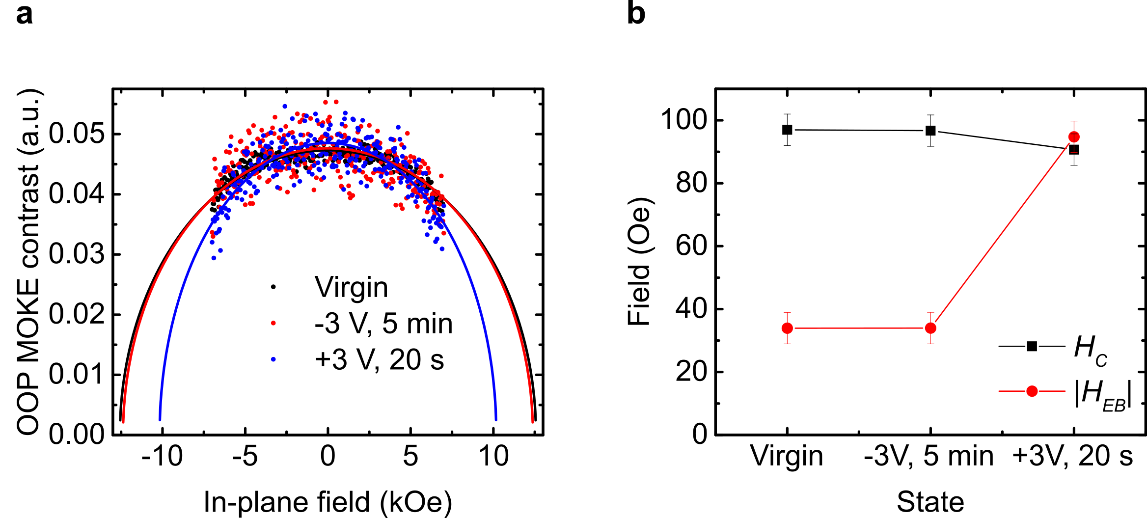


**Figure S12. a,** Out-of-plane (OOP) MOKE contrast as function of in-plane field for three different states. Solid lines are fits to Eq. S7. **b**, Coercivity and EB for the same states as that in **a**. Error bars represent typical variation observed during hysteresis loop measurement.

**11. Speed of H-loading in multilayer films:**

We performed experiments to determine the rate of H-loading in the multilayer films as a function of gate voltage. Fig. S13a shows the percent change in EB from the initial value as a function of time for different applied potentials. Sizable change in the EB can be detected for $V_{G}$ ≥ 1 V, indicating that, in this system, the minimum potential to dissociate atmospheric water vapor and drive hydrogen into the magnetic layers is around 1 V.


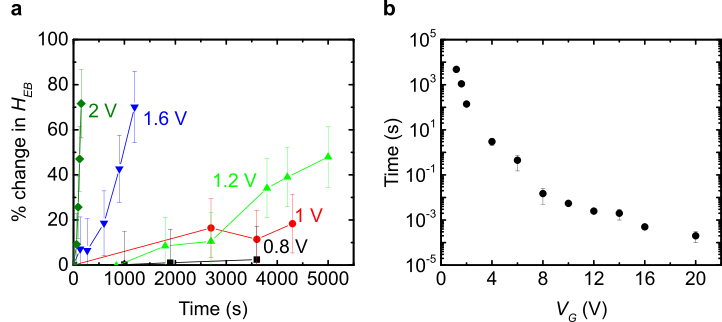


**Figure S13. Speed of H-loading. a,** Change in EB (as a percentage of initial value) as a function of time for various applied potentials. Error bars are derived from typical variation observed during hysteresis loop measurement. **b,** Time required to increase EB by at least 50% as a function of gate voltage. Error bars represent the difference between the shortest accumulative gating time to observe >50% increase and longest accumulative gating time to observe <50% increase in EB. Relative humidity was >50% in all cases while ambient and sample temperature were around 22 ^o^C.

From experiments like that of Fig. S13a, the time to 50% increase in EB can be estimated and plotted as a function of applied potential, as shown in Fig. S13b. Sub-millisecond timescales are reached at potentials higher than 14 V. Even faster rates are expected for heterostructures tailored for speed. H-extraction cannot be performed at such high speeds, the reasons for which are not entirely clear. Possible factors include secondary reactions taking place at high negative bias, lateral diffusion of H out of the device region or some other factor. These aspects will be addressed in greater detail in future work.

**References:**

1. Xi, H. & White, R. M. Theory of the blocking temperature in polycrystalline exchange biased bilayers based on a thermal fluctuation model. *J. Appl. Phys.* **94**, 5850–5853 (2003).

2. Grimes, R. W. & Lagerlöf, K. P. D. Polymorphs of Cobalt Oxide. *J. Am. Ceram. Soc.* **74**, 270–273 (1991).

3. Rooksby, H. P. Structure of Nickel Oxide. *Nat. 1943 1523854* **152**, 304–304 (1943).

4. Ehresmann, A., Schmidt, C., Weis, T. & Engel, D. Thermal exchange bias field drift in field cooled Mn 83 Ir 17/Co 70 Fe 30 thin films after 10 keV He ion bombardment. *J. Appl. Phys.* **109**, 23910 (2011).

5. Carey, M. J. & Berkowitz, A. E. Exchange anisotropy in coupled films of Ni81Fe19 with NiO and CoxNi1−xO. *Appl. Phys. Lett.* **60**, 3060–3062 (1992).

6. Markin, T. L. & Dell, R. M. Recent developments in nickel oxide-hydrogen batteries. *J. Electroanal. Chem. Interfacial Electrochem.* **118**, 217–228 (1981).
